# Qualitative Analysis of Surveyed Emergency Responders and the Identified Factors That Affect First Stage of Primary Triage Decision-Making of Mass Casualty Incidents

**DOI:** 10.1371/currents.dis.d69dafcfb3ad8be88b3e655bd38fba84

**Published:** 2016-08-19

**Authors:** Kelly R. Klein, Frederick M. Burkle Jr., Raymond Swienton, Richard V. King, Thomas Lehman, Carol S. North

**Affiliations:** UT Southwestern Medical Center at Dallas; Harvard Humanitarian Initiative, Harvard University, Cambridge, MA, USA; University of Texas Southwestern Medical Center; University of Texas Southwestern Medical CenterUniversity of Texas Southwestern Medical Center; Co-Director National Training Center-WestNDLS Foundation; ProfessorThe Altshler Center for Education & Research at Metrocare Services and The University of Texas Southwestern Medical Center, Department of Psychiatry

**Keywords:** Disaster medicine, Emergency management, Mass casualty care, Triage

## Abstract

Introduction: After all large-scale disasters multiple papers are published describing the shortcomings of the triage methods utilized. This paper uses medical provider input to help describe attributes and patient characteristics that impact triage decisions.

Methods: A survey distributed electronically to medical providers with and without disaster experience. Questions asked included what disaster experiences they had, and to rank six attributes in order of importance regarding triage.

Results: 403 unique completed surveys were analyzed. 92% practiced a structural triage approach with the rest reporting they used “gestalt”.(gut feeling) Twelve per cent were identified as having placed patients in an expectant category during triage. Respiratory status, ability to speak, perfusion/pulse were all ranked in the top three. Gut feeling regardless of statistical analysis was fourth. Supplies were ranked in the top four when analyzed for those who had placed patients in the expectant category.

Conclusion: Primary triage decisions in a mass casualty scenario are multifactorial and encompass patient mobility, life saving interventions, situational instincts, and logistics.

## INTRODUCTION

A mass casualty incident (MCI) is defined as an event which generates more patients at one time than locally available resources can manage using routine procedures.**** 1 The goal of triage systems, protocols and algorithms, at every level of care, is to ensure the best possible opportunity for survival of all the victims served. Advancements within emergency medical services (EMS) have had considerable impact on the manner in which MCIs are triaged and initially managed.

Primary triage occurs at the first contact with the EMS medical personnel at which point victims are assigned an acuity level based on injury severity. Secondary triage, or a reevaluation of the victim’s condition after initial medical care, may also occur at the scene of the MCI following EMS interventions or during transport to an emergency department or secondary collection station.^2^ The decision-making processes involved in primary triage and patient hospital distribution are influenced by both reactive (ad hoc) and proactive (based on situational awareness) factors.^2^ An ‘ideal’ triage protocol system would result in minimal under-triage or patients classified less acute than what they really are, and over-triage, which has been shown to increase mortality as more people are labeled as a higher acuity than they really are.

To date, no single triage tool algorithm can demonstrate sufficient scientific evidence to justify national adoption. In 2006 the National Association of EMS Physicians (NAEMSP) and the Centers for Disease Control and Prevention (CDC) funded the SALT workgroup. The goal was to exam published triage systems and make recommendations based on available science for the adoption of one standard mass casualty triage system. The initial result of the workgroup effort showed that there was no published triage system that could be adopted. Secondary outcomes were two fold; the first was the development a new triage system, the Sort-Assess-Lifesaving Interventions-Treatment/Triage (SALT). This a non-proprietary free system developed from available research, with widely accepted best practices of existing mass triage systems, and consensus opinion from the workgroup. The second outcome, because of resistance from local, state and federal agencies to change current triage practices which, would allow interoperability among existing triage tool algorithms, the SALT workgroup, developed the Model Uniform Core Criteria (MUCC) for Mass Casualty Triage. The MUCC consists of 24 criteria of recommended elements of a MCI triage system (Table 1).

**Figure:**
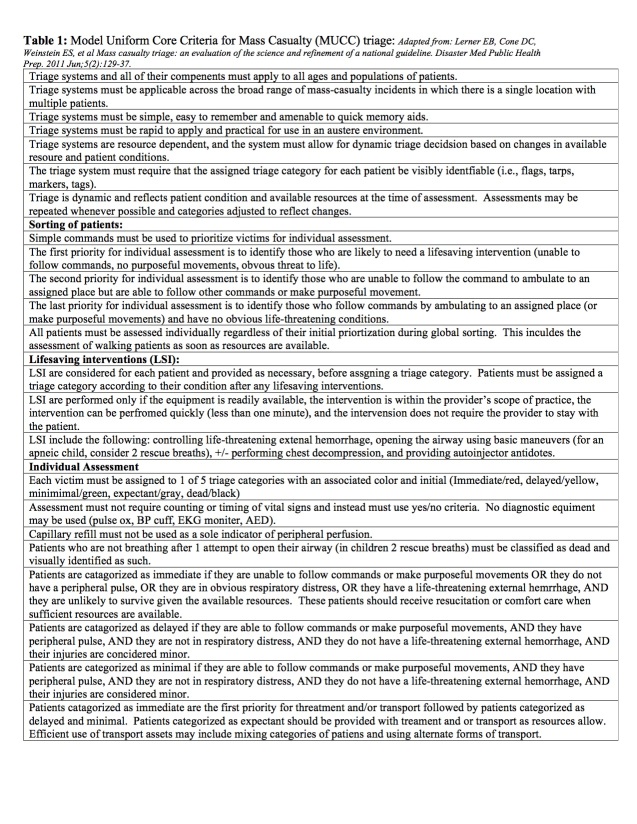
**Table 1: Model Uniform Core Criteria for Mass Casualty (MUCC) triage**

The criteria, if adopted into existing triage systems, would allow more for uniformity and interoperability between EMS responders from different jurisdictions upon arrival at an MCI site. As of 2011, 15 of the 24 MUCC essential elements are being used by existing triage systems; SALT is totally compliant with all 24 criteria.**** 3

In recognition that current classification systems may not be accurate enough or properly mitigate subjectivity, several studies including those by ethicists and critical care specialists have suggested that, due to social, medical, and logistical variables, that only an experienced provider or a triage team approach should be utilized during the most difficult triage decisions.**** 4 ^,^ 5 ^,^ 6 Additionally, very few studies have satisfactorily identified, compared, and discussed the actual real time decision-making criteria used by experience-rich providers, in order to create an accurate and usable prioritization algorithm for mass casualty events.**** 7 ^,^ 8 ^,^ 9 ^,^ 10

To better understand both the reactive and situational awareness factors influencing the primary triage decision-making process among pre-hospital responders, both experienced and not, this study was designed to investigate and analyze those factors that actual medical providers during MCIs perceived that directly and indirectly impacted and influenced how their primary triage priority decisions were processed.

## METHODS

To evaluate how triage decisions are initially processed, this study distributed an electronic survey to EMS and pre-hospital medical providers to help identify and rank the factors that influence primary triage decisions made by those with and without disaster experience. The survey questionnaire was designed by the authors, in English, to ascertain what attributes healthcare workers value and utilize when evaluating a patient for triage and treatment priority. The initial survey was piloted with a small sample of ten healthcare workers who work in an academically affiliated emergency department and have had previous disaster response experience. Based upon the feedback received, the final survey instrument was revised and the modified questions were entered into SurveyMonkey®. The survey was available on-line for 2 months (April-May 2010). The survey was then distributed electronically via a variety of methods: personal email addresses to disaster responders, emergency medicine and disaster listservs, and a hyperlink on a medical website frequented by prehospital EMS medical professionals (http://www.jems.com/articles/2010/05/mci-triage-techniques-survey.html). Respondents were not provided any financial or other incentives for participation. The survey study received approval by our hospital’s institutional review board prior to the start of data collection. Surveys that were not completed in their entirety were excluded from the final analysis.

****Survey Instrument:****

The introductory paragraph described the intent of the survey, provided assurance of anonymity, and indicated that the approximate time to complete the survey would be less than 20 minutes (this was based on Beta testing of the survey on SurveyMonkey®). By survey design, all questions were required to be answered and the respondent could not advance without answering; The only exception was for specific stand-alone questions asking for opinion-based write-ins, if these were not answered, the respondent’s survey was still included.

General information required, aside from date of birth and gender included:

- Primary professional role (e.g., physician, nurse, EMT (emergency medical technician, Paramedic)
- Medical specialty (e.g., emergency medicine, prehospital care)
- Years in medical profession (dichotomized as 10 or fewer years vs. >10 years)
- Disaster training if any (military, NDLS, ARC, FEMA, decontamination, CDP-Noble, or other)
- Triage system used in both non-disaster and disaster practice (ESI, CDP-Noble, MASS, SAVE, START, SALT, STM informal “gut feeling”, or other)
- Disaster triage experience (yes/no)
- Experience of triaging a “live” patient to an expectant category (yes/no)
- Specific disaster deployments if applicable

Patient attributes contributing to triage placement: Six patient and disaster attributes were to be ranked in the order of importance to the respondent (1-6 with 1 being the most important); to help that would determine the preferred ranking of these attributes by the respondent.

- Ability of the patient to speak to you
- Age
- Gut feeling, by the responder performing triage, of the severity of injury
- Perfusion-peripheral pulse
- Respiratory status
- Supplies or resource availability

In addition, for those who claim prior disaster response experience, a comment box was added to allow for additional observations to be added besides the six listed attributes and/or factors that might influence the triage priority of patients.

## RESULTS

**Data Analysis**

A total of 495 surveys were returned, with 92 deemed incomplete and thusly excluded from data analysis. The 403 completed surveys were analyzed using SAS 9.3 for Windows. Descriptive findings are presented as numbers, proportions, means, frequencies, and standard deviations. Respondents, for further analysis, were classified into three groups based on their disaster triage experience: no disaster triage experience (*NoExp*), performance of disaster triage without the “expectant” category (*ExpNoExpectant*) experience, and performing disaster triage with an “expectant” category, those people who are not expected to survive based upon logistical and or skill limitations. Those with “expectant category” (*ExpBT*) experience (n=47) were classified as the most triage experienced respondents of the three groups. Wilcoxon (non-parametric) tests compared mean rankings between the different triage experience groups. Bonferroni adjustments to the alpha level were made for 12 comparisons, and for these comparisons, alpha level was set at p<.004 (.05/12). Chi-square statistics were used to compare the dichotomous top four vs. lowest two rank order of the supplies/resource availability factor and of the patient age factor between the *NoExp* vs. *ExpNoExpectant* groups and between the *ExpNoBT* and *ExpBT* groups.

Fifty three percent of the 403 respondents were pre-hospital providers (Table 2).

**Figure:**
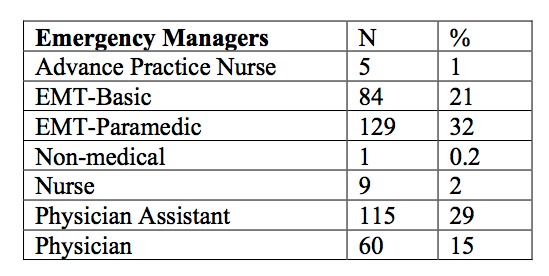
**Table 2. Self reported medical occupations of respondents, all of who worked in emergency management areas. (N=403)**

Nearly all indicated that they practiced a structured triage approach (i.e., START, MASS, SAVE, STM, ESI) with 8% (32/403) reporting that they utilized an informal “gut feeling” triage methodology. The *NoExp* group comprised the majority, 60% (240/403), 29% (116/403) were in the *ExpNoBT* group, and 12% (47/403) were in the *ExpBT* group. Those in both the *ExpNoBT* and *ExpBT* groups, were able to expound on their personal disaster experiences. These included deployments to military conflicts, Oklahoma City bombing, 9/11 for both World Trade Center and Pentagon sites, and protracted disaster events such as hurricanes Katrina and Ike.

The mean (SD) ranking of each of the six-triage factors by the three experience groups is displayed in Figure 1.

**Mean rankings of triage factors by respondent's disasters experience level.**
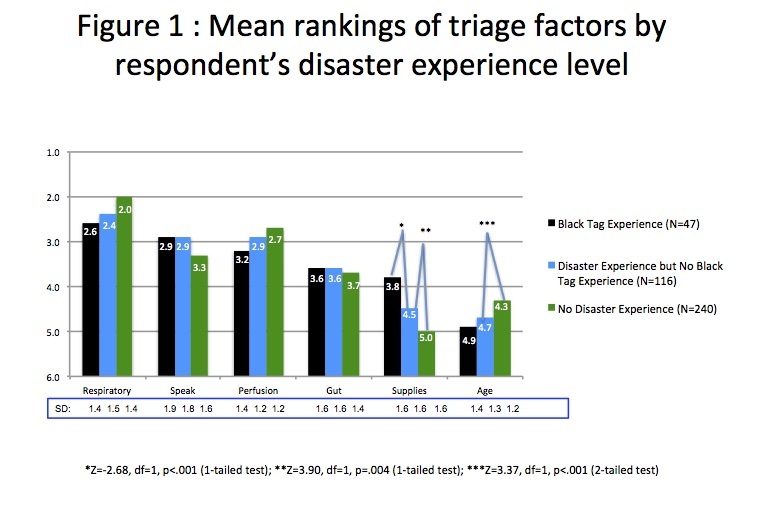

‘Respiratory status, ability to speak, perfusion/pulse, and gut feeling’ factors were ranked in the top four by the majority of the 403 respondents. The mean rankings for five of the six variables (all but ‘gut feeling’) differed by experience groups and were all statistically significant depending on disaster experience (Table 3).

**Figure:**
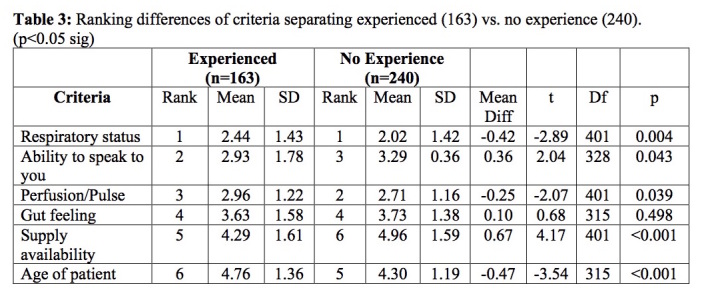
**Table 3: Ranking differences of criteria separating experienced, black tag with and without disaster experience (163) vs. no disaster experience (240). (p<0.05 sig)**

Though there were slight differences in the order of ranking, both groups had the same top and bottom three criteria choices with ‘supplies and age’ showing strong significance in their ranking placement differences. Further analysis with a series of nonparametric Wilcoxon tests comparing the factor rankings across each of the disaster experience group pairs found the following significant between-group comparisons: the *NoExp* group ranked the ‘supplies/resource availability’ factor significantly lower than did the *ExpNoBT* group who in turn ranked the ‘supplies/resource availability’ factor significantly lower than did the *ExpBT* group. The Z proportion of each of the three experience groups ranking ‘supplies/resource availability’ in the top four is demonstrated in Figure 2.

**Percent of respondents by triage experience category, who ranked 'supplies' in the top four choices.**
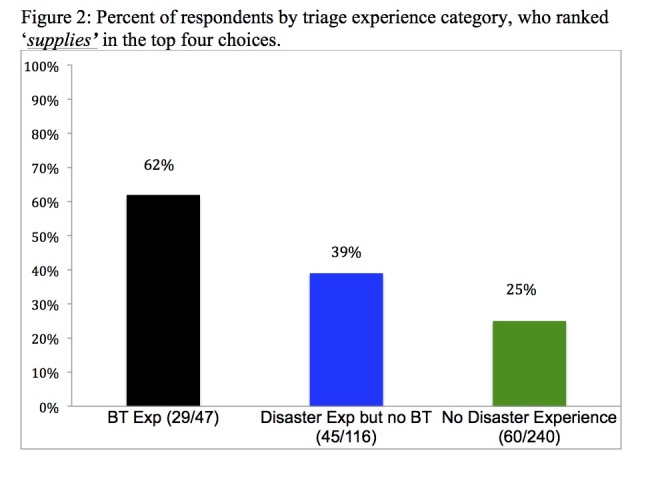

The *NoExp* group had a significantly lower Z proportion ranking the ‘supplies/resource availability’ factor in the top four than in the *ExpNoExpectant* group (c2=7.2, df=1, p=.008), who in turn had a significantly lower proportion, ranking the ‘supplies/resource availability’ factor in the top four, than the *ExpExpectant* group (c2=7.1, df=1, p=.008). This statistical significance is important when reviewing resource utilization (‘supplies/resource availability’) considerations among the three groups. The *ExpBT* group employed ‘resource utilization’ based factors as important in disaster triage decision-making 62% of the time compared with *ExpNoBT* and *NoExp* groups, who employed it 39% and 25% of the time, respectively.

## LIMITATIONS

Surveys are not strong science, with a sampling bias in this case due to the use of specific and limited access listservs, personal email lists, and web site visit. The results were analyzed as professionally pooled and therefore might be skewed toward the larger numbers of physicians, EMTs, and paramedics among the EMS providers surveyed. The electronic survey limited the pool of respondents to those willing and able to use and access the survey using a computer. The true potential response rate is unknown as the actual number of people who received an invitation via email list or listserv is unknown. The use of a convenience sample, such as we used, did not allow for us to have any control over who chose to respond. It is not an ideal method of gathering data, but in this case, it allowed for the survey to be distributed to a much larger geographic population to give a better sense of the triage provider population at large and was not cost prohibitive, as we only had to pay for the SurveyMonkey® annual fee of $400 U.S.. In addition, the top three ranked attributes (airway, circulation, and neurological status) are seen in START triage, possibly skewing their priority ranking due to the Hawthorne affect, where participants change behavior because they are aware of being observed. Moreover, the survey did not inquire whether triage decisions were made individually, or with assistance such as a team and this triage aspect will be included in subsequent studies.

## DISCUSSION

Only in a perfect world would the ideal MCI triage system exist that accurately identified each casualty by severity and type of injury/illness, ensures that victims received the proper treatment and transport prioritization, and are delivered to appropriate medical care quickly and efficiently. Admittedly, in every mass casualty event patients who are able to leave the scene often do so often prior to EMS arrival. Those remaining are either dead, need an intervention to save their life, are still on scene because they cannot ambulate, or are rending bystander medical assistance; as was witnessed during the Boston Marathon bombing. Those victims left on scene, as organized first responders arrive, are evaluated, and often moved to a gathering location where more medical supplies or shelter is available (i.e. treatment tent in the Boston Marathon, I-10 causeway, Louis Armstrong international airport). It is here that transportation and other logistical considerations become additional factors that may impact triage decisions, all of which are an integral part of pre-hospital decisions. Interestingly, aside from SALT, no popular triage algorithm discusses patient movement, transport, and logistics.

Ideally, triage decisions, made without emotional input or bias, should be able to identify those victims likely to survive if taken to definitive care. However, the ranking of victims into severity and priority treatment categories makes triage dynamic and, depending on numerous external variables, can be an emotive activity that might skew priority categories, possibly introducing bias, leading to either under triage or over triage, which, as mentioned earlier, can cause an increase in mortality. Recently, Cross and Cicero compared six different triage systems and found that none worked well for every event with most resulting in over triage.**** 11 Travers and colleagues suggest that triage systems providing the most reliable and effective patient outcomes have multiple triage categories, with the optimum number of five levels felt to be “safer, with greater discrimination, reliability and improved specificity”.**** 12 This was independently supported by others such as Burkle whose triage levels include: immediate, delayed, minimal, expectant, and deceased as recommended for bioevent triage, and with ICU teams where physician seniority was considered a favorable independent factor in ICU admission criteria.**** 5 ^,^ 13

Admittedly, triage is a fluid and dynamic process; it has multiple variables influencing the decision maker and should be adaptable to a myriad of situations. Most triage criteria endorsed by the American College of Surgeons, Emergency Nursing Association, and the American College of Emergency Physicians use a combination of physiological, anatomical, mechanism of injury, and special considerations categories to determine severity categorization.^14^^,^ 15 It is created as a step-by-step algorithm for the provider to follow to allow for the best patient transportation designation. However, triage is a very complex task that is dynamic, heuristic, and driven primarily by “provider judgment rather than specific triage criteria”.^16^ Newgard and colleagues further demonstrated the “independent predictive value” of the EMS provider’s judgment, a gut feeling-like primary triage criteria for identifying seriously injured victims, showing that the “cognitive reasoning processes encapsulated in this criteria can help in identifying seriously injured patients potentially missed by other triage criteria.”^17^

The purpose of this survey was to query practicing field experts and identify factors that for them affect their triage category placement of patients. The assumption made is that medical providers with practical experience in triage situations will prioritize different attributes for the sorting of patients compared to those providers with no disaster experience. These findings in turn, could provide more attribute weighting of the factors used create a more accurate triage algorithm. For example, in this study, the top ranked categories regardless of experience level were ‘cardiovascular’ and ‘neurological’ with those respondents with disaster experience placing a higher value on the ability of the patient to ‘speak’ than on the ‘cardiovascular’ criteria. This suggests that brain perfusion to the experienced triage provider is considered a good indicator of injury severity.

After the physiological attributes, in our rankings, ‘Gut instinct' ranked forth, regardless of disaster experience level with 8% reporting, that they used it as their primary form of triage guidance. Instinct or gut instinct is related to experience, which is a part of expert knowledge, and is very effective in cases of complex decisions such as triage.**** 18 In the nursing literature, much has been written about experience and instinct in patient care, with an acceptance that experience creates ‘intuition’ which guides recognition of subtle deterioration and subsequent appropriate nursing action.**** 19 In the prehospital literature the support of ‘gust instinct’ has been mixed. A review of the earlier prehospital triage literature is varied, with some studies suggesting that provider judgment is effective in increasing the sensitivity of triage.**** 16 ^,^ 20 ^,^ 21 ^,^ 22 Other papers suggest it has no affect at all, which could be due to a lack of overall provider experience.**** 23 ^,^ 24 There have been two large retrospective trauma studies examining prehospital triage and destination assignment to determine what historically prehospital providers have used for decision-making. These analyzed a three year period where >250,000 injured person were transported; 36% of the time, EMS provider judgment was the most commonly used triage criterion.**** 17 ^,^ 25 This is further supported in a variety of published case reports which have noticed that ‘gut instinct’ played an enormous role in correctly assigning triage criteria to patients.**** 16 ^,^ 17 ^,^ 18 ^,^ 25 ^,^ 26

Supplies ranked 5th amongst the 403 factors analyzed, in toto, was found to rank in the top three in 68% of responses when subset analysis was performed on those claiming prior disaster experience. Other than being used in the SACCO scoring system, this factor is not part of the most popular triage algorithm decision trees, but is part of the MUCC criteria and SALT system.**** 27 When the write-in sections were reviewed, respondents with Expectant Category experience “wrote in” that the reason for the placing of patients in the expectant category was due to the lack of ‘supplies/transportation’. This pivotal point is not unique to the pre-hospital or disaster arena. It has been described often in the critical care literature, where, during times of resource limitations in critical care units, patients who normally would meet admission/treatment criteria are refused a bed in favor of patients with a perceived more favorable set of sociological and medical parameters that might predict a better outcome.**** 26 ^,^ 28 ^,^ 29 ^,^ 30

This document supports the MUCC, as it provides an additional framework for the development of local and adaptable triage algorithms that include resource availability, sorting, lifesaving interventions, and individual patient assessment endorsing five triage categories. From the MUCC came the resulting CDC endorsed SALT triage (Figure 3)

**SALT Triage:**
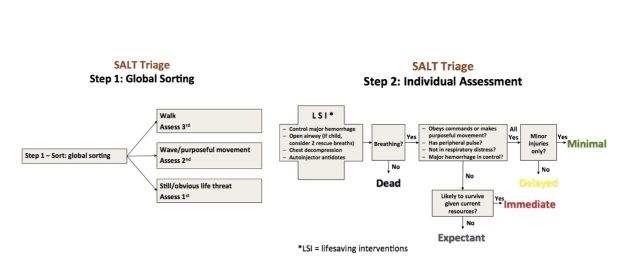

as an example of a disaster triage algorithm and is presently taught as part of the National Disaster Life Support (NDLS) course series. Triage is a fluid and dynamic process; it has multiple variables influencing the decision maker and should be adaptable to a myriad of situations

## CONCLUSION

Based upon this survey analysis, respondents both with and without direct disaster triage experience identified and ranked triage attributes that support the MUCC guidelines. What appears to best support a balanced disaster mass casualty triage system and considered the most important factors among the surveyed respondents are ‘neurological/cardiovascular’ condition, ‘resource availability’, and the personal attribute of ‘gut instinct’. In addition, decision making in primary triage of a MCI scenario is multifactorial and encompasses life saving interventions, patient mobility, situational instincts and logistics all considered as critical components of a triage scheme that needs more study and analysis. Consensus places experience during MCIs as very important. Based on the success of this survey, the designed electronic survey tool is considered reliable for a second stage international EMS qualitative analysis study of factors influencing primary triage decisions.

## Competing Interests

The authors have declared that no competing interests exist.

## Data Statement

All relevant data are available within the paper.

## Abbreviations

ARC: American Red Cross

CDP-Noble: Center for Domestic Preparedness-Noble Training Center

ESI: Emergency Severity Index

FEMA: Federal Emergency Management Agency

MASS: Move Assess, Sort, Send

NDLS: National Disaster Life Support

SALT: Sort, Assess, Lifesaving Interventions, Treatment/Transport

SAVE: Secondary Assessment of Victim Endpoint

START: Simple Triage and Rapid Treatment

STM: Sacco Triage Method
